# Remarks on Wisdom Teeth

**Published:** 1859-07

**Authors:** Abr. Robertson

**Affiliations:** Wheeling, Va.


### REMARKS ON WISDOM TEETH.

BY ABR. ROBERTSON, D. D. S., M. D.

MESSRS. EDITORS: In accordance with my promise, I herewith send you a few hastily written, though long considered, observations on some of the lesions occasioned by the eruption of the wisdom teeth, and on the best method of removing that class of teeth.

That the wisdom teeth produce many and very serious esions, is abundantly evident without the citation of any cases. I have myself had most convincing experimental personal proof of the fact in two well-marked cases—my own two lower wisdom teeth. And I studied their history, progress and character with *most intense interest*, at intervals longer or shorter, and more or less frequent, for three, or two years at least; and I have been observing their effects in others now these twenty years. But it was shown in your last number that it is not by "lateral pressure" that the wisdom or any other teeth give us pain or any other serious cause of complaint. How, then, do they so affect us? In various ways. First, as all other teeth do, or at least are liable to do, by the pressure (generally perpendicular, but may be horizontal or diagonal, the direction of the pressure being entirely immaterial) of the tooth upon the gum in its effort to force its way through it. The pressure spoken of, being that necessarily produced by the formation and development of the tooth; sometimes causing inflammation, pain, etc.

Sometimes, when the tooth comes through the gum in a horizontal or in a diagonal direction, with its grinding—or, in such cases, more properly its multi-cuspidated—surface against the cheek, (I suppose I ought to say buccal muscles, or the mucous membrane lining the inner surface of the buccal muscles, instead of plain cheek,) if the cusps happen to be sharp or a little rough, irritation and inflammation may be produced by attrition.

Sometimes, again, when the space between the jaws is small, and the tooth in one jaw—the antagonist to the one now coming—has already acquired its full length, and, as occasionally happens by the coming tooth being backward in its coming, more than its proper length, and therefore occupies more than its due proportion of the limited space between the jaws, the gum is so far raised by the coming tooth as to bring it in contact with its antagonist, so that at every occlusion of the jaws it gets bruised between the opposing teeth, and thus, temporarily, a good deal of discomfort is produced.

Again, finally, and by far the most frequently, and worst of all, where, as in the preceding case, the space between the jaws is less than the length of the crowns of two teeth, or where one tooth by long priority in coming has taken to itself more than its due proportion of the space designed to be equally divided between the two, room enough is not left for the projection of the entire crown of the other through the gum. And as the gum can form no attachment to the crown of a tooth—the enamel having no periosteum—it remains a kind of open sack or pouch about the tooth, as deep as the unprotruded portion of its crown. Sometimes there is only room for a very small portion of the crown to come through the gum, even to only one or two of its points. Then the sack has a depth equal to the whole length of the crown of the tooth. This forms a ready receptacle for food, saliva, or any other substances taken into or secreted by the mouth, where they may be retained till they are decomposed and produce irritation more or less, which may result in inflammation, ulceration, the burrowing of pus deep in the cellular tissue, forming abscess, causing necrosis, exfoliations, and all the other ills consequent upon severe inflammation. These more serious lesions, so far as my observation goes, are confined to the lower jaw; and for the reason that, I believe, the upper wisdom teeth are usually first in coming, and are therefore most fully protruded through the gum; but chiefly for the reason that if the food or other substances were forced by mastication or otherwise, under the edge of the gum on the upper jaw, the force of gravitation would tend to remove it, and would generally do so before it could do much harm; whereas, in the lower jaw the same force would tend to keep it there.

In regard to treatment, very little need be said; for if the crowns of the opposing teeth have already met before they are fully protruded through the gums, and especially if the lower tooth is, and can be, but partially protruded, the only effectual remedy that I have ever found is the removal of the tooth; thus obliterating the sack and preventing a recurrence of the trouble. But where the trouble is only the irritation or slight inflammation, produced by the tooth forcing its way through the gum, an incision, or at most a slight excision, of the gum may be all that is necessary to afford entire relief. But, as from this cause the discomfort is usually but temporary, even this or any other treatment is comparatively unimportant. But since extraction, in most of the cases that are of any consequence, is the only remedy to be relied on, the best means of removing the wisdom teeth becomes a question of very serious importance.

A correspondent of the *Dental News Letter* for April last, asks if any one knows a better way of extracting the wisdom teeth, where the jaws were so nearly closed by swelling occasioned by the eruption of such a tooth, that he could not get his mouth open "but a little" *after forcing it with a wedge*, than by the use of the turnkey! Allow me to say, that I can not conceive of a worse way. I had supposed—and do suppose—that the key had been laid aside by all well-educated dentists long ago, and that now it was only to be found among the antiquarian collections of the bygone days of barbarity; or at most only among the instruments of some few "old-fogy" operators who do everything just as they were taught to do it, and continue to do it so just because they were taught to do it so; who still twist their foil into little ropes or strings preparatory to inserting it into the cavities of teeth; who take all their impressions in "pure yellow beeswax;" and who still use zinc for making the dies with which they swage their mouth-plates. But even allowing the key to be—what I do not allow—an appropriate and useful instrument, in some cases, or even for certain classes of teeth, as some, perhaps, still claim for it, the anatomy of the teeth and jaws, both, most clearly forbid its use, even under the most favorable circumstances, in the extraction of the lower wisdom teeth; and a more barbarous operation than attempting to extract a wisdom tooth with a key, when the face is so much swelled as so nearly to close the mouth as to leave only enough room between the teeth to allow the hook of the instrument to pass, and only by the use of some small instrument to aid in pushing it along, at that, as described in the article "on extracting wisdom teeth" before alluded to, I can not readily imagine; for it is perfectly evident that the only action that the instrument could possibly have, under the circumstances, would be either to break the tooth or to tear away the alveolus. And if breaking off the crown of the tooth, or of removing a portion of the alveolus was the object in view, that end could be far more easily and safely attained than by wrenching them off with a canthook or turnkey.

But there is another question in the short article alluded to, not less ill-considered than this—"What was to be done then? Take out a sound tooth to make room for the forceps, or resort to some other instrument?"

Is it not plain that if, from any cause, the jaws are inseparably closed and the arch of the teeth unbroken, the wisdom tooth is the only one that can possibly be removed except by violently breaking off its crown. Take out a sound tooth to get at the wisdom tooth, indeed!

But, as I said in relation to "*lateral pressure,*" this would not be worthy of any consideration, except for the *quasi*-endorsement it receives by being published in a respectable dental journal, and without comment by its editors.

I have said that anatomy forbids the use of the key, especially in the extraction of the lower wisdom teeth. It does so, first because a very large share—a very large majority, I think—of the roots of the lower wisdom teeth are more or less hook-shaped, or at least approximating to the form of the segment of a circle; sometimes, indeed, a large portion of the root stands almost at a right-angle to the crown, and the foramina, as in all other cases, looking toward the ramus of the jaw.

Now it is evident that the turnkey can not be applied to a tooth shaped as described and situated like the wisdom teeth, in the very back part of the mouth, so as to apply force in the direction required easily or safely to remove it.

The force of the key, when properly applied—if there can be any proper application of an improper thing—is upward and inward or upward and outward, as the case may be; whereas the only force that can act directly for the removal of such a tooth, without certainly breaking the tooth or the surrounding bone, must be upward and *backward.*

But not only the anatomy of teeth but of the jaws, render the turnkey, and not only that, but the forceps too, but poorly adapted to the extraction of that class of teeth. At that part of the jaw there is but little, if any, alveolar process, and the jaw is broad, and nearly flat upon its top, so that there is no opportunity to apply either the hook of the key or the beaks of the forceps any lower than just at the base of the crown, with an almost certainty of breaking off or of crushing the tooth. When forceps are used in the extraction of any molar tooth, the points of its beaks should always enter the bifurcation of its fangs in order to act with certainty or with safety. This can but rarely be accomplished on the wisdom teeth.

Their situation in the mouth, too, sometimes renders the application of either of these instruments difficult. The narrow space, also, between the upper and lower jaws sometimes also prevents the application, with safety to the mouth, of sufficient force to remove the tooth with the forceps, even if they could be otherwise properly adjusted.

For these reasons, for many years past, I have but very rarely attempted to extract any lower wisdom tooth, if the tooth next front of it had not already been removed, with any other instrument than the elevator. The one I use is a modification of the common straight elevators of the shops; being bent backward in the shaft just behind the spear-like shaped heads at an angle of about twenty degrees. This form greatly facilitates its introduction between teeth so far back in the mouth, and also adds very much to its power.

I scarcely need say, that the instrument is used by inserting its point freely and firmly between the necks of the tooth to be removed and the one next front of it, using the front one of the two—second molar—as a fulcrum for the instrument. By giving it a slightly rotary motion, it acts as a powerful lever, and exerts its force in the exact direction required to remove teeth situated and shaped as these usually are. The wisdom teeth can be removed, too, with this instrument, without difficulty, with the jaws closed to any extent short of absolute occlusion. It is a powerful instrument, and of course, like all other instruments, requires care and skill to use it properly.

*Wheeling, Va.*, June, 1859.

---

THE D. M. C. of the Ohio Dental College, says that he has discovered a new remedy for the treatment of DENTAL PUPS, which will knock thunder out of the "Eastern Liquid Quarts," four to the gallon. A. P.

